# The *Legionella pneumophila* Effector Protein, LegC7, Alters Yeast Endosomal Trafficking

**DOI:** 10.1371/journal.pone.0116824

**Published:** 2015-02-02

**Authors:** Kevin M. O’Brien, Elizabeth L. Lindsay, Vincent J. Starai

**Affiliations:** 1 Department of Microbiology, University of Georgia, Athens, Georgia, Unites States of America; 2 Department of Infectious Diseases, University of Georgia, Athens, Georgia, United States of America; University of Louisville, UNITED STATES OF AMERICA

## Abstract

The intracellular pathogen, *Legionella pneumophila*, relies on numerous secreted effector proteins to manipulate host endomembrane trafficking events during pathogenesis, thereby preventing fusion of the bacteria-laden phagosome with host endolysosomal compartments, and thus escaping degradation. Upon expression in the surrogate eukaryotic model *Saccharomyces cerevisiae*, we find that the *L. pneumophila* LegC7/YlfA effector protein disrupts the delivery of both biosynthetic and endocytic cargo to the yeast vacuole. We demonstrate that the effects of LegC7 are specific to the endosome:vacuole delivery pathways; LegC7 expression does not disrupt other known vacuole-directed pathways. Deletions of the ESCRT-0 complex member, *VPS27*, provide resistance to the LegC7 toxicity, providing a possible target for LegC7 function *in vivo*. Furthermore, a single amino acid substitution in LegC7 abrogates both its toxicity and ability to alter endosomal traffic *in vivo*, thereby identifying a critical functional domain. LegC7 likely inhibits endosomal trafficking during *L. pneumophila* pathogenesis to prevent entry of the phagosome into the endosomal maturation pathway and eventual fusion with the lysosome.

## Introduction

*Legionella pneumophila* are ubiquitous aquatic bacteria and obligate intracellular pathogens that infect a variety of phylogenetically diverse aquatic amoebae and protists [1, 2]. *Legionella* are opportunistic pathogens of humans, able to infect and invade human alveolar macrophages if contaminated water is aerosolized and inhaled, causing a severe form of pneumonia known as Legionnaires’ disease, as well as the milder, self-limiting infection, Pontiac fever [3, 4].

*Legionella pneumophila* requires a type IVb secretion system (Dot/Icm; defective in organelle trafficking/intracellular multiplication) for intracellular survival [5], which allows the translocation of nearly 300 known and predicted effector proteins into the host cell [6–9]. Many of these proteins are thought to directly disrupt normal host membrane trafficking pathways in order to both prevent the lysosomal degradation of *Legionella*, and to promote the synthesis of the specialized intracellular replicative niche termed the Legionella-containing vacuole (LCV) [10–12]. The LCV is composed of both plasma membrane and ER membrane components, which requires both a major diversion of ER-derived vesicles from normal trafficking pathways, and the aberrant SNARE protein-dependent fusion of those membranes [12–15].

A number of the *Legionella* effector proteins contain motifs with high similarity to eukaryotic proteins, and are thought to function by manipulating eukaryotic host cell events by mimicking or modulating host proteins. [16–18]. Some of the effectors thought to directly alter host cell membrane trafficking events contain coiled coil motifs (LegC) including LegC2, LegC3, and LegC7 [11, 12, 19]. LegC7/YlfA was originally identified as a *Legionella* effector protein that resulted in cell death upon expression in the budding yeast *Saccharomyces cerevisiae* [19]. It was also found that expression of LegC7 resulted in vesicular accumulations on the yeast vacuole and aberrant secretion of CPY-Invertase, inducing an apparent a yeast class E vacuolar protein sorting (VPS) phenotype [11, 19, 20]. As there is a high degree of conservation amongst genes involved in cellular transport and fusion across eukaryotic biology, these studies provided essential information into the function of LegC7/YlfA during *Legionella* pathogenesis.

The yeast endosomal trafficking pathway serves as an important hub that links the processes of endocytosis and vacuole-directed biosynthetic traffic; vesicles derived from the Golgi or plasma membrane fuse to establish early endosomes that undergo a conserved maturation process, which ultimately concludes with the fusion of late endosomes with the degradative vacuole (reviewed in [21]). To solve the topology “problem” in the degradation of integral membrane proteins, the yeast multivesicular endosome/body (MVB) is a specialized late-stage maturing endosome characterized by the presence of intraluminal vesicles (ILVs) that contain membrane proteins bound for degradation in the yeast vacuole [22]. ILVs are formed due to the action of a highly conserved protein-sorting complex called the endosomal sorting complex required for transport (ESCRT) complex (reviewed in [23]), which functions by recognizing and packaging ubiquitin modified membrane proteins into ILVs for degradation in the vacuole lumen [24, 25]. Deletion of many of the ESCRT genes, or class E VPS genes, results in a malformed MVB and aberrant secretion of CPY-Invertase, a normally vacuolar directed protein [20, 26, 27].

As expression of LegC7 results in an apparent class E phenotype in yeast cells, we hypothesized that LegC7 exerts its toxic effect at some point in the endosomal trafficking pathway and that likely one or more of the class E genes are required for the toxicity of LegC7. Herein, we show that deletion of the yeast ESCRT-0 gene, *VPS27*, results in a decrease in LegC7 toxicity. Furthermore, we see that LegC7 causes a severe disruption of both vacuole-directed biosynthetic traffic and endocytic cargo pathways, while not disrupting alternative vacuolar transport pathways. Localization to, and formation of, class E compartments, disruption of both biosynthetic and endocytic traffic, and genetic interaction with an ESCRT protein all indicate that LegC7 functions to modulate endosomal traffic. These data help provide a deeper understanding of LegC7 function in eukaryotic cells.

## Materials and Methods

### Yeast strains and plasmid construction

Yeast strain SEY6210 (MATα *his3-Δ200 trp1-Δ901 leu2–3,112 ura3–52 lys2–801 suc2-Δ9*) was used for GFP-Vam3 and Ste3-GFP localization studies. Yeast strain BY4742 (MATα *his3*Δ*1 leu2*Δ*0 lys2*Δ*0 ura3*Δ*0*) was used for all other studies, and a full list of strains and plasmids used are included in Table 1.

**Table 1:** Strains and plasmids used in this study.

Strain	Genotype	Source
BY4742	MATα *his3*Δ*1 leu2*Δ*0 lys2*Δ*0 ura3*Δ*0*	[60]
SEY6210	MATα *his3-Δ200 trp1-Δ901 leu2–3,112 ura3–52 lys2–801 suc2-Δ9*	[26]
BY4742 *vps27∆*	BY4742 *vps27∆::KANMX6*	GE Healthcare Dharmacon
BY4742 *hse1∆*	BY4742 *hse1∆*::KANMX6	GE Healthcare Dharmacon
BY4742 *vps27Δ hse1∆*	BY4742 *vps27∆*::KANMX6 *hse1∆::NATMX*	This Study
BY4742 *srn2∆*	BY4742 *srn2∆::KANMX6*	GE Healthcare Dharmacon
BY4742 *vps36∆*	BY4742 *vps36∆::KANMX6*	GE Healthcare Dharmacon
BY4742 *snf8∆*	BY4742 *snf8∆::KANMX6*	GE Healthcare Dharmacon
BY4742 *vps25∆*	BY4742 *vps25∆::KANMX6*	GE Healthcare Dharmacon
BY4742 *vps20∆*	BY4742 *vps20∆::KANMX6*	GE Healthcare Dharmacon
BY4742 *vps24∆*	BY4742 *vps24∆::KANMX6*	GE Healthcare Dharmacon
BY4742 *did4∆*	BY4742 *did4∆::KANMX6*	GE Healthcare Dharmacon
BY4742 *vps4∆*	BY4742 *vps4∆::KANMX6*	GE Healthcare Dharmacon
BY4742 *vps28∆*	BY4742 vps28*::KANMX6*	GE Healthcare Dharmacon
BY4742 *snf7∆*	BY4742 *snf7∆::KANMX6*	GE Healthcare Dharmacon
BY4742 *bro1∆*	BY4742 *bro1∆::KANMX6*	GE Healthcare Dharmacon
BY4742 *vps23∆*	BY4742 *vps23∆::KANMX6*	GE Healthcare Dharmacon
BWY640	SEY6210 *vam3∆*::HIS3 pPRS306 GFP-GFP-Vam3	[58]
BWY2858	SEY6210 Ste3-GFP::KAN	[33]
BWY3400	SEY6210 *ent1Δ*::LEU2 *ent2*ΔHIS3 *yap1801*Δ::HIS3 *yap1802*Δ::LEU2 Ste3-GFP::KAN +pBW0778[pRS414::*ent1*(aa1–151)]	[33]
Plasmid	Characteristics	Source
pVJS52	pYES2/NT C, *legC7*, *ura3*	[12]
pVJS53	pYES2/NT C, *legC7, lys2*	This Study
pVJS54	pYES2/NT C, *legC7N242I, ura3*	This Study
pVJS55	pYES2/NT C, *legC7N242I, lys2*	This Study
pGO36	pRS416, URA3	[29]
pMM134	pRS416, Sna3-GFP	[30]
pGO45	pRS416, GFP-CPS	[29]
pVJS47	pTYB12 LEGC7ΔTM	[12]
pVJS56	pET42a, GST-VPS27	This Study
pVJS57	GFP-Vps27 (pGO36)	This Study
pMM2660	*ura3* to *lys2* converter	GE Healthcare Dharmacon
pVJS59	GFP-LegC7(pGO36)	This Study

The creation of a galactose inducible yeast expression vector in pYES2/NTc for *LEGC7* expression, pVJS52, was previously described [12]. For some experiments, the *URA3* locus on this plasmid was converted to *LYS2* via standard lithium acetate transformation methods and homologous recombination with HindIII-digested pM2660 [28], resulting in pVJS53.

To create GFP-LegC7 expression vectors via gap repair, *LEGC7* was amplified using primers GFPC7_R and GFPC7_F, and the resulting amplicon was co-transformed with linearized pGO36 plasmid (a generous gift from Dr. Alexey Merz, University of Washington-Seattle) [29] into BY4742, creating pVJS59. GFP-Vps27 was created in the same manner, using primers GFPVps27_F and GFPVps27_R.

A plasmid for the purification of GST-Vps27 was constructed by amplifying *VPS27* from BY4742 genomic DNA using primers Vps27BamHI_F and Vps27XhoI_R, digested with BamHI and XhoI, and ligated into plasmid pET-42a (Novagen) digested with the same enzymes, resulting in pVJS56.

### Recombinant protein purification

LegC7∆TM protein was purified as previously described [12], except that the final elution from chitin beads was performed for 48 h at 22°C. Eluted protein was dialyzed into PS buffer (20mM PIPES-KOH, pH 6.8, 200mM sorbitol) containing 300mM KCl. Antibodies against LegC7ΔTM were raised in rabbits using a standard protocol and subsequent serum used for Western blots at a 1:5000 dilution (Rockland Immunochemicals, Inc).

GST-Vps27 was purified by standard glutathione affinity chromatography, using pVJS56 as the expression vector. Eluted protein was dialyzed into PS buffer containing 150mM KCl.

### Random mutagenesis of *LEGC7*

In order to locate regions of *LEGC7* required for toxicity in yeast, BY4742 harboring pVJS52 was grown in selective media at 30°C for 18 h, and 1 OD_600_ unit was harvested by centrifugation. This pellet was washed with 1M sterile sodium phosphate buffer, pH 7.0, suspended in 1mL of the same buffer, and the chemical mutagen ethyl methanesulfonate (EMS) was added to 2% (v/v). After shaking at 30°C for 1 h, 1 mL of 1M sodium thiophosphate was added, and the cells were plated directly to CSM-uracil plates containing 2% galactose. Plasmids were isolated from each resulting colony. These plasmids were re-introduced into BY4742; plasmids that were no longer inhibitory, but expressed full length LegC7 by immunodetection, were sequenced (Georgia Genomics Facility, University of Georgia).

Site-directed mutagenesis of pVJS52 was carried out via standard PCR techniques with the following primer pairs: C7N242_F/C7N242_R (LegC7^N242I^), C7N242D_R/C7N242D_F (LegC7^N242D^), C7N242Q_R/C7N242Q_F (LegC7^N242Q^), C7N242L_F/C7N242L_R (LegC7^N242L^), C7N242A_F/C7N242A_R (LegC7^N242A^), or C7N242R_R/C7N242R_F (LegC7^N242R^) (Table 2).

**Table 2:** Primers used in this study.

Primer Name	Sequence*
LegC3-KpnI	5′-GTAGAA*GGTACC*CGTGATTATGTTTTTGGCCAAC-3′
LegC3-XbaI	5′-GGTGGT*TCTAGA*GCTCCATTGAAATTTTATTGACAG-3′
GFPC7_F	5′-ATGGATGAACTATACAAGTCCGGACTCAGATCTATGGCTACTAATGAAACAG-3′
GFPC7_R	5′-GCTTTAGTCAATTAAAGATCTCGAGCTCAAGCTTCGAATTCTGCAGTCGAC-3′
C7N242_F	5′-CTGATTTATTGGAAAAAATTCAAAAGGAATTGTCAAAA-3′
C7N242_R	5′-CTGATTTATTGGAAAAAATTCAAAAGGAATTGTCAAAA-3′
Vps27BamH1_F	5′-GGAGGA*GGATCC*GACAGTATGTCCGTTAGCACGCC-3′
Vps27Xho1_R	5′-GGAACTGCTAATAGAGCTTTAATA*CTCGA*GGGAGGA-3′
C7N242Q_F	5′-CTGATTTATTGGAAAAACAACAAAAGGAATTGTCAAAA-3′
C7N242Q_R	5′-CTGATTTATTGGAAAAACAACAAAAGGAATTGTCAAAA-3′
C7N242R_F	5′-CTGATTTATTGGAAAAACGTCAAAAGGAATTGTCAAAA-3′
C7N242R_R	5′-CTGATTTATTGGAAAAACGTCAAAAGGAATTGTCAAAA-3′
C7N242D_F	5′-CTGATTTATTGGAAAAAGATCAAAAGGAATTGTCAAAA-3′
C7N242D_R	5′-CTGATTTATTGGAAAAAGATCAAAAGGAATTGTCAAAA-3′
C7N242L_F	5′-CTGATTTATTGGAAAAACTTCAAAAGGAATTGTCAAAA-3′
C7N242L_R	5′-CTGATTTATTGGAAAAACTTCAAAAGGAATTGTCAAAA-3′
C7N242A_F	5′- CTGATTTATTGGAAAAAGCTCAAAAGGAATTGTCAAAA-3′
C7N242A_R	5′- CTGATTTATTGGAAAAAGCTCAAAAGGAATTGTCAAAA-3′
Vps27GFP_F	5′-ATGGATGAACTATACAAGTCCGGACTCAGATCTATGTCCGTTAGCACGCCAAG-3′
Vps27GFP_R	5′-GGAACTGCTAATAGAGCTTTAAAGATCTCGAGCTCAAGCTTCGAATTCTGCAGTCGAC-3′

*Italics denote introduced restriction sequences

### Lucifer Yellow uptake assay

Cells were grown for 18 hours at 30°C in CSM-uracil with 2% glucose, collected by centrifugation, washed with sterile water, suspended in CSM-uracil with 2% galactose and grown for 16 hours at 30°C. 1.0 OD_600_ unit of cells were harvested by centrifugation and suspended in 100μL fresh CSM-uracil containing 2% galactose. Lucifer Yellow was added to 8mg/mL and samples were incubated for 2 h at 30°C. Ice-cold Lucifer Yellow stop buffer (50mM potassium phosphate, pH 7.5, 10mM sodium azide) was added with mixing, and cells were pelleted. Samples were washed 3 additional times with Lucifer Yellow stop buffer to ensure removal of extracellular dye. Samples were suspended in 100μL Lucifer Yellow stop buffer, mixed with an equal volume of a 0.6% agar solution, and mounted for fluorescence microscopy.

## Results

### LegC7 N242I is no longer toxic when expressed in yeast

**Figure 1:**
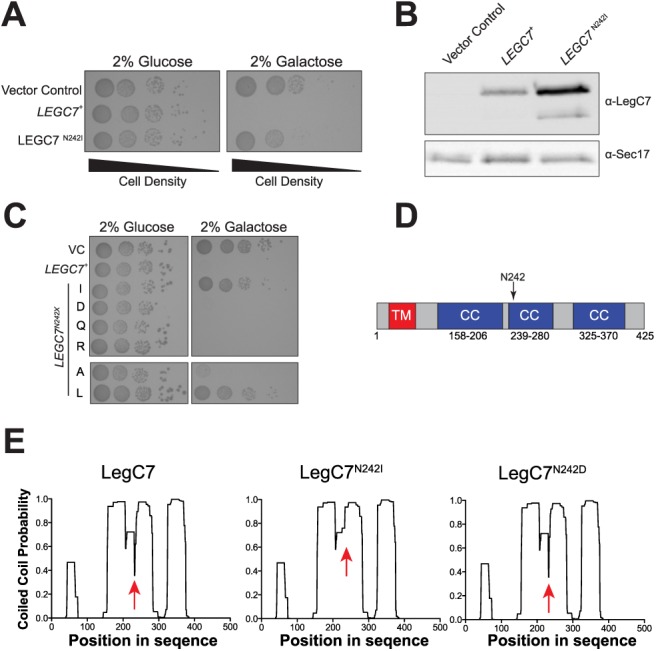
Residue N242 is required for LegC7 toxicity in yeast. (A) BY4742 yeast strains harboring the galactose-inducible control plasmid pYES2/NT C, pVJS52 (*LEGC7* ^+^), or pVJS54 (*LEGC7* ^*N242I*^) were spotted onto CSM-uracil medium supplemented with either 2% glucose or 2% galactose with 10-fold serial dilutions from a starting culture of OD_600_ = 1.0. Plates were incubated for 72 h at 30°C. (B) Strains from (A) were grown in for 24 h in CSM-uracil supplemented with 2% glucose at 30°C, washed in ddH_2_O, suspended in fresh CSM-uracil/2% galactose, and incubated at 30°C for 16 h. Equal fractions of each strain were harvested, total protein was extracted [55], and 30μl from each sample was separated by SDS-PAGE. Samples were immunoblotted for LegC7 (rabbit 1:5000) or Sec17p (Rabbit, 1:1000) [56] (loading control). (C) The *LEGC7* ^+^ plasmid, pVJS52, was mutagenized via site-directed mutagenesis (Materials and Methods), transformed into BY4742, and spotted onto CSM-Ura medium containing either 2% glucose or 2% galactose in 10-fold serial dilutions. (D) Diagram of the predicted LegC7 protein structure indicating transmembrane domain (TM, red) and three predicted coiled coil domains (CC, blue). Transmembrane prediction was calculated with TMHMM Server v.2.0 (http://www.cbs.dtu.dk/services/TMHMM/,) and coiled coil predictions were calculated with COILS (http://toolkit.tuebingen.mpg.de/pcoils) with a window size of 21, weighting, and an iterated matrix. (E) Coiled coil probability prediction of LegC7 containing either N, I, or D at position 242 were run as in (D). Probabilities at each position were plotted and the predicted disordered region between predicted coiled coil regions 1 and 2 is marked (red arrow).

As LegC7 expression is known to be toxic upon expression in yeast [19], identification of residues critical for this activity *in vivo* would likely be important for understanding the mechanism of LegC7-mediated toxicity. Therefore, we mutagenized yeast containing a galactose-inducible *LEGC7* plasmid using ethyl methanesulfonate (EMS). Plasmids that were no longer inhibitory were sequenced, and one plasmid contained a single nucleotide transversion at position 725 that produced a mutant protein substituting an isoleucine for asparagine at amino acid position 242 (LegC7^N242I^). This single nucleotide transversion strongly reduced the toxicity of *LEGC7* expression *in vivo* (Fig. 1A); immunoblots confirmed that LegC7^N242I^ was expressed to levels similar to (or greater than) LegC7 expression (Fig. 1B).

In attempt to dissect the function of N242 in LegC7, we used site directed mutagenesis to introduce a number of other amino acids into this position, including a conservative change (N242Q), charged residues (N242R and N242D), and hydrophobic residues (N242A or N242L). Interestingly, only mutation of N242 to hydrophobic residues (N242I or N242L) resulted in abrogation of LegC7 toxicity; N242A shows a very slight reversal phenotype (Fig. 1C). This particular residue (N242) is predicted to be one of the first residues of the second putative coiled coil region, based on *in silico* models (Fig. 1D). Just prior to the second coiled coil domain of LegC7, probability models of coiled coil structure predict a sharp decrease in coiled coil domain formation probability (Fig. 1E, left panel, red arrow). When replacing asparagine 242 with isoleucine, however, this *in silico* model predicts that the disordered region is eliminated (Fig. 1E, middle panel, red arrow); replacing N242 with a residue that did not reduce LegC7 toxicity showed coiled coil probabilities similar to the wild type protein (Fig. 1E, right panel, red arrow). Based on these coiled coil prediction methods, asparagine 242 may be essential for the proper folding of either the second coiled coil domain or small loop region just upstream of this domain. A previous report identified that this coiled coil domain is essential for LegC7 toxicity, but was based on large deletions in this region [19]. Therefore, we have identified a single residue in LegC7 responsible for LegC7 function *in vivo*.

### LegC7 disrupts endosome to vacuole traffic

**Figure 2:**
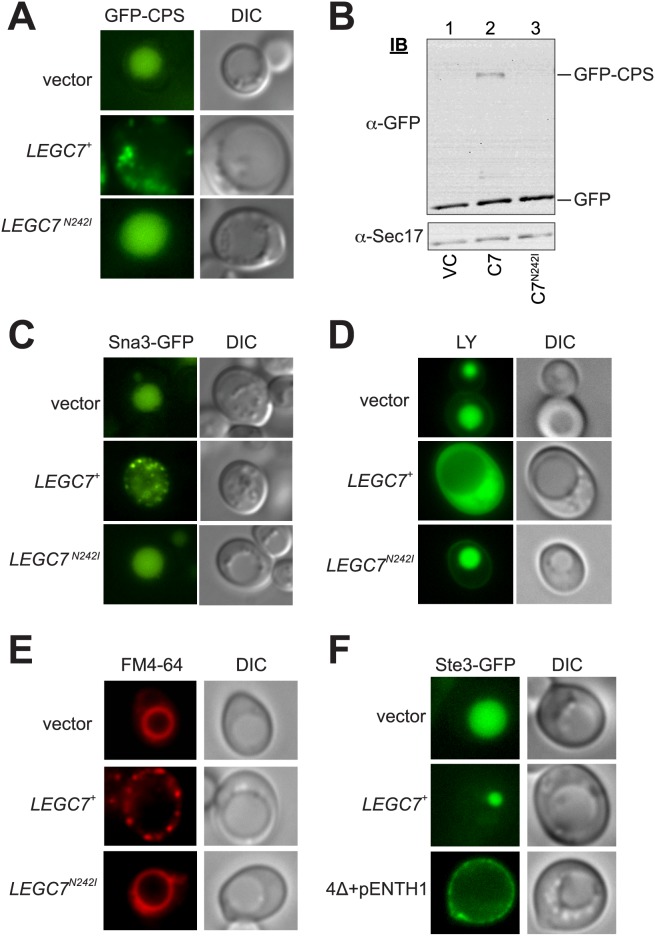
LegC7 induces endosome:vacuole trafficking defects. (A) BY4742 yeast strains harboring GFP-CPS and either the vector control, *LEGC7* ^+^, or *LEGC7* ^*N242I*^ plasmids were grown in selective media supplemented with 2% glucose at 30°C, washed in ddH_2_O, suspended in fresh CSM-uracil/2% galactose, incubated at 30°C for 16 h, then visualized. (B) Equal portions of total proteins were extracted from strains in (A), then immunoblotted for GFP and Sec17p (loading control). (C) BY4742 yeast strains containing GFP-Sna3 and either the vector control, *LEGC7* ^+^, or *LEGC7* ^*N242I*^ plasmids were grown as in (A), then visualized. (D) Cells containing the vector control, *LEGC7* ^+^, or *LEGC7* ^*N242I*^ plasmids were incubated with Lucifer Yellow (Materials and Methods), and then visualized. (E) Strains from (D) were grown in selective media supplemented with 2% glucose at 30°C, washed in ddH_2_O, suspended in fresh CSM-uracil/2% galactose, incubated at 30°C for 16 h, then stained with the yeast vacuolar marker FM4–64 [57] and visualized. (F) Wild type SEY6210 or ∆4+ENTH (Table 1) strains harboring Ste3-GFP and either the vector control or *LEGC7* ^+^ plasmids were grown as in (A) and then visualized.

It is known that expression of LegC7 in yeast induces a vacuolar protein-sorting defect based upon the observed mis-sorting and extracellular secretion of a vacuole-directed CPY-invertase fusion protein when *LEGC7* ^+^ is expressed [11]. In order to further characterize the protein sorting defects induced by LegC7 in yeast, we examined the endosomal trafficking patterns of several well-defined yeast proteins upon *LEGC7* ^+^ expression. Carboxypeptidase S (CPS) is a vacuolar protease known to traffic to the vacuole via the CPY (Golgi-endosome-multi-vesicular body) route [29]. Accordingly, yeast cells harboring GFP-tagged CPS protein in the absence of LegC7 show a distinct localization of GFP-CPS to the vacuole lumen (Fig. 2A). Upon expression of *LEGC7*, however, GFP-CPS is strongly localized to the cell periphery in a diffuse punctate pattern (Fig. 2A, S1A Fig.). In confirmation that the LegC7^N242I^ protein is no longer active *in vivo*, cells expressing LegC7^N242I^ deliver GFP-CPS to the vacuole lumen, as in wild type strains (Fig. 2A, S1A Fig.).

In order to confirm delivery of GFP-CPS was disrupted in strains expressing LegC7, we took advantage of the fact that the GFP-CPS protein is known to be cleaved upon delivery to the proteolytic vacuole, resulting in an easily-detectable size shift upon immunoblot for GFP [30]. Strains expressing LegC7 show a clear accumulation of the full length GFP-CPS fusion protein (Fig. 2B). In contrast, strains harboring the inactive LegC7^N242I^ protein or vector control plasmid show complete conversion of the GFP-CPS protein to the lower molecular weight GFP (Fig. 2B), confirming the observation that delivery of GFP-CPS to the vacuole is drastically altered in *LEGC7* ^+^ strains (Fig. 2A).

In order to examine LegC7’s effect on the delivery of another well characterized membrane protein to the vacuole, we examined the trafficking of the Sna3p protein, which is sorted to the MVB membrane via its close association with, and ubiquitination by, the ubiquitin ligase Rsp5p [31]. Sna3p is then packaged into ILVs at the MVB and localized to the vacuole lumen upon MVB:vacuole fusion [30]. As expected, Sna3-GFP was localized to the vacuole lumen in yeast strains lacking LegC7 (Fig. 2C). Expression of LegC7, however, resulted in the striking accumulation of Sna3-GFP in either peripherally-localized punctae, or in a diffuse cytosolic staining pattern (Fig. 2C, S1B Fig.). Expression of LegC7^N242I^, does not affect normal vacuolar localization of Sna3-GFP (Fig. 2C, S1B Fig..), confirming that this mutant derivative of LegC7 has lost *in vivo* function.

LegC7 expression is known to induce a class E phenotype in yeast upon expression, leading to vacuole protein sorting defects and aberrant protein secretion of biosynthetic traffic through the MVB [11]. Therefore, we hypothesized that LegC7 may also induce defects in the delivery of endocytic cargo to the vacuole. In order to assay for endocytic defects in the presence of LegC7, we measured the ability of yeast to accumulate the soluble fluorescent dye, Lucifer Yellow (LY). LY is known to enter yeast via endocytosis, and is delivered to the vacuole where it accumulates [32]. Under galactose growth conditions, yeast accumulates LY in the vacuole, as expected (Fig. 2D). In contrast, strains expressing LegC7 fail to accumulate LY in the vacuole, but rather display a cytoplasmic accumulation phenotype (Fig. 2D, S1C Fig.); LegC7^N242I^ does not block endocytic delivery of LY to the vacuole (Fig. 2D, S1C Fig.). Furthermore, when we attempted to stain the yeast vacuolar membrane of LegC7-expressing strains with the fluorescent styryl dye, FM4–64, we observed accumulations of the dye in punctate structures lining the plasma membrane, staining not seen in either vector control or LegC7^N424I^-expressing strains, highly reminiscent of the structures seen to accumulate GFP-CPS and Sna3-GFP (Fig. 2E, S1D Fig.). These results strongly suggest a defect in endocytic delivery of FM4–64 to the vacuole in strains expressing LegC7. Recent work from our laboratory has shown normal FM4–64 staining patterns of the vacuole in LegC7-expressing strains [12], however those images were taken three hours post-induction. After a 16h galactose induction, strains harboring LegC7 display these clear defects in FM4–64 delivery to the vacuole. Taken together, these data show that LegC7 either delays or inhibits normal endosomal traffic to the vacuole, from both biosynthetic and endocytic pathways.

In order to examine the effects of LegC7 on the disruption of receptor-mediated endocytosis, we utilized the a-factor pheromone receptor, Ste3p, fused to GFP [33]. Without the appropriate ligand, Ste3 is constitutively endocytosed and delivered to the vacuole [34]. Under galactose growth conditions Ste3-GFP accumulates in the vacuole as expected (Fig. 2F). When LegC7 is expressed, however, Ste3-GFP accumulates in a single, small compartment on the vacuolar periphery, reminiscent of a class E compartment (Fig. 2F, S1E Fig.). In order to more explicitly define whether LegC7 prevents the endocytic uptake of Ste3-GFP, we utilized a strain with the 4 clathrin-binding adaptor proteins (Ent1p, Ent2p, Yap1801p, and Yap1802p) deleted and complimented with an epsin N-terminal homology domain (4Δ + pENTH1), a strain previously shown to be defective in the uptake of Ste3-GFP from the plasma membrane via endocytosis [33, 35]. In this strain background, Ste3‐GFP accumulated at the plasma membrane as expected (Fig. 2F). The clear distinction between the plasma membrane accumulations of Ste3-GFP in the strain defective for endocytic uptake (4∆ + pENTH1) and the strain expressing LegC7 show that LegC7 does not disrupt the physical process of endocytosis, but rather prevents the proper vacuolar delivery of the endocytosed Ste3-GFP cargo.

In order to determine if the disruption in endosomal trafficking was due simply to LegC7-induced cell death, we stained cells with propidium iodide (PI) which is only internalized upon cell membrane disruption after cell death [36]. We found that after the standard 16 hour galactose induction period, cells expressing the vector control plasmid showed that approximately 94% of the cells excluded PI, compared to nearly all of the yeast cells subjected to excess heat (S2A Fig.). Strains expressing LegC7 show PI staining levels indistinguishable from vector control strains, suggesting that the observed endosome trafficking defects are not due to widespread cell death induced by LegC7 (S2A Fig.).

### LegC7 does not inhibit endosome-independent traffic to the vacuole

The yeast vacuole receives cargo from at least 3 pathways (Reviewed in [37]): the endocytic/vacuolar protein-sorting pathway (CPY pathway) [20], directly from the Golgi in an AP-3 adapter complex-dependent manner (ALP pathway) [38], and directly from the cytosol via autophagic processes [39]. Of these three pathways, however, only the endocytic/CPY pathway utilizes endosomal intermediate vesicles for vacuolar delivery, and we therefore sought to measure the effects of LegC7 on vacuolar trafficking pathways that do not require endosomal intermediates.

**Figure 3:**
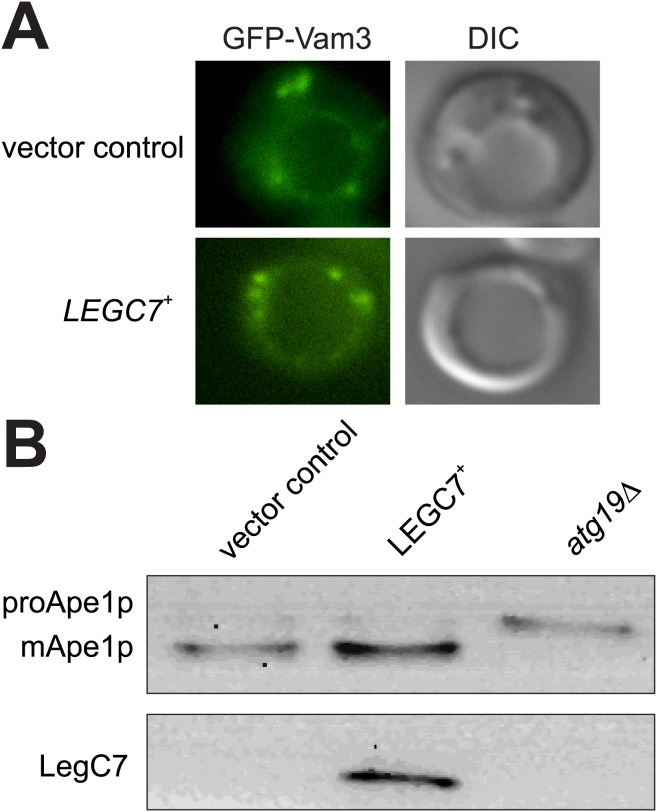
LegC7 does not delay non-endosomal vacuolar traffic. (A) Wild type yeast strains expressing GFP-Vam3 [58] and expressing either *LEGC7* ^+^ or *LEGC7* ^*N242I*^ were grown in selective media supplemented with 2% glucose at 30°C, washed in ddH_2_O, suspended in fresh CSM-uracil-lysine/2% galactose, incubated at 30°C for 16 h, then visualized. (B) Wild type or *atg19∆* cells expressing either *LEGC7* ^+^ or *LEGC7* ^*N242I*^ were grown in selective media containing 2% glucose at 30°C, washed in ddH_2_O, suspended in fresh CSM-uracil/2% galactose, incubated at 30°C for 16 h, and total proteins were extracted from equal fractions. Proteins were separated and immunoblotted for Ape1p (Rabbit 1:2000) [39] and LegC7.

The vacuolar SNARE, Vam3p, traffics directly from the Golgi to the vacuole through the AP-3/ALP pathway; no interaction with the endocytic pathway is observed [40]. Yeast strains expressing GFP-Vam3p show a clear localization of Vam3p to the vacuolar membrane (Fig. 3A). Strains expressing LegC7 show no obvious defect in GFP-Vam3 trafficking (Fig. 3A, S2B Fig.), in contrast to our previous data showing disruption of known endosomal traffic from the Golgi to the vacuole (Fig. 2A,C).

As a marker for the delivery of cytosolic components to the vacuole via a specialized autophagic process known as cytosol-to-vacuole targeting (Cvt), we measured the maturation of the vacuolar aminopeptidase, Ape1p. This protein is produced in a cytosolic proenzyme form, selectively encapsulated by an autophagosomal membrane, and delivered to the vacuole for proteolytic processing and enzymatic activation [39]. This processing can be easily observed via immunoblot, and wild type yeast shows the expected maturation of the Ape1p polypeptide, confirming normal Cvt trafficking. Expression of LegC7 does not disrupt the maturation of Ape1p while strains lacking Atg19p, the Ape1 receptor required for proper Ape1 delivery to the vacuole, result in an accumulation of unprocessed precursor (Fig. 3B) [41, 42]. These data indicate that the inhibitory effects of LegC7 on trafficking pathways are specific to endosomal traffic, and not the result of general trafficking or vacuolar defects.

### Deletions in *vps27* reduce LegC7 toxicity

Previous reports have indicated that low-level expression of LegC7 results in the formation of so-called “class E” compartments, and that LegC7-GFP is localized to these compartments [11]. Furthermore, LegC7 was reported to induce the aberrant secretion of CPY-Invertase, a protein that should be directed to the vacuole via the CPY pathway [11, 19]. As these results phenocopy known class E trafficking mutants in yeast, we hypothesized that one or more class E VPS genes may be required for LegC7 toxicity, even though deletions of individual class E genes did not cause major disruptions in LegC7-GFP localization [11].

**Figure 4:**
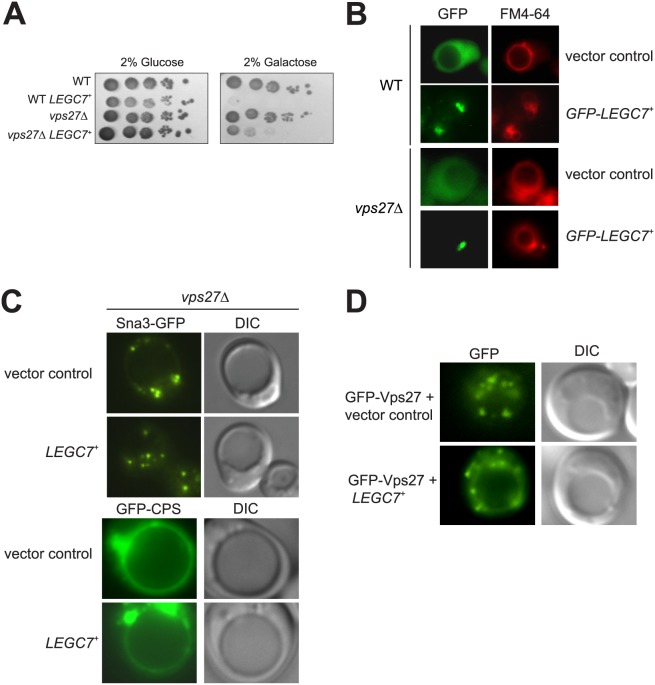
Deletion of *VPS27* reduces LegC7 toxicity. (A) BY4742 or *vps27∆* strains harboring either the control or *LEGC7* ^+^ plasmids were spotted onto CSM-Ura plates containing 2% glucose or 2% galactose in 10-fold serial dilutions (starting OD_600_ = 1.0) and grown at 30°C for 96h. (B) BY4742 or *vps27∆* strains expressing GFP or GFP-LegC7 were grown in selective media supplemented with 2% glucose at 30°C, stained with FM4–64, and visualized for GFP and FM4–64 fluorescence. (C) Yeast *vps27∆* strains expressing either GFP-CPS or Sna3-GFP harboring the *LEGC7* ^+^ expression plasmid or vector control were grown in selective media supplemented with 2% glucose at 30°C, washed in ddH_2_O, suspended in fresh CSM-uracil-lysine/2% galactose, incubated at 30°C for 16 h, then visualized. (D) Cells expressing GFP-Vps27 and LegC7 were grown as in (C), and localization of GFP-Vps27 was determined.

We assayed LegC7 toxicity in each of the single deletions of the 13 originally-identified class E mutants (*vps4∆, vps20∆, vps23∆, vps24∆, vps25∆, vps27∆, vps28∆, vps36∆, bro1∆, snf7∆, snf8∆, srn2∆,* and *did4∆*) [20] and *hse1∆*. These genes define many of the proteins that comprise the various ESCRT complexes and accessory factors which are required for both the biogenesis of the MVB, and in the proteolytic turnover of ubiquitinated proteins; their functions in this pathway is highly ordered process. Interestingly, deletion of only the ESCRT-0 member *vps27* resulted in a partial reversal of toxicity of LegC7 (Fig. 4A); no other single class E gene deletion affected LegC7 toxicity (S3 Fig.).

As a member of the ESCRT-0 complex, Vps27p is a multifunctional protein that binds ubiquitinated proteins, binds to endosomal phosphatidylinositol 3-phosphate (PI3P) via its FYVE (Fab-1, YOTB, Vac1, and EEA-1) domain, and recruits the ESCRT-I complex to the endosome via direct interactions with Vps23p [43–45]. ESCRT-0 functions as 1:1 heterodimer of Vps27p and Hse1p [46, 47]. Surprisingly, the *hse1∆* single deletion strain did not reduce the toxicity of LegC7 (S3 Fig.), and the double deletion strain (*hse1∆ vps27∆*) did not show any additional reversal of LegC7 toxicity beyond the effect of the *vps27∆* deletion (SI3). Given that *hse1∆* strains are not resistant to LegC7, nor are any ESCRT mutants downstream of Vps27 function, the *vps27*-mediated reversal of LegC7 toxicity is likely due to a specific function, and not a direct result of defective ESCRT complex activity.

As Vps27p functions to target ubiquitinated membrane proteins bound for vacuolar turnover, we hypothesized that suppression of LegC7 toxicity in *vps27∆* strains may be due to either mislocalization of LegC7, or altered proteolytic turnover of LegC7 *in vivo*. GFP-LegC7 localizes to vesicular accumulations reminiscent of class E compartments, confirming a previous report (Fig. 4B, [11]). The localization of GFP-LegC7 is not drastically altered in Δ*vps27* strains, which is also consistent with this report (Fig. 4B, S4A Fig. [11]). Given that ESCRT complex proteins are important in regulating membrane protein turnover, we measured LegC7 turnover in *vps27∆* strains. After 60 minutes of incubation, LegC7 is near undetectable in wild type yeast extracts (S4B Fig.). In *vps27∆* strains, however, there appears to be less LegC7 present at all timepoints taken (S4B Fig.), when compared to wild type. Therefore, LegC7 levels in *vps27∆* strains are reduced either through enhanced proteolytic turnover, or through a reduction in expression. Interestingly, Sec18p, a protein that should degrade over the course of this assay [48], also appears to degrade more quickly in *vps27∆* strains, while Sec17p remains stable over the assay in both strains (S4B Fig.). Therefore, it appears that some proteins may turnover more quickly in *vps*27∆ strains, and lower levels of LegC7 *in vivo* may explain the resistance of *vps27∆* strains to LegC7 expression.

It is known that *vps27∆* strains have defects in delivery of endosomal traffic to the vacuole, presumably due to an aberrant MVB function [20]. When we observed the trafficking of the endosomal cargoes Sna3-GFP and GFP-CPS in *vps27∆* strains, we noted the expected punctate trafficking defect of Sna3-GFP, similar to that seen in LegC7-expressing strains (Fig. 4C, S4C and D Fig., Fig. 2C). Additionally, GFP-CPS accumulated on the vacuolar membrane in *vps27∆* strains, and was not delivered to the vacuolar lumen (Fig. 4C, S4D Fig.). To measure whether or not LegC7 and Vps27p interact directly, we purified GST-Vps27p and attempted to pull-down recombinant LegC7∆TM [12]. We did not detect an interaction between GST-Vps27 and LegC7ΔTM *in vitro* (S5A Fig.), although either the transmembrane domain or short N terminal region of LegC7 could be required for interaction of these two proteins, or LegC7∆TM may require ubiquitination for this interaction to occur. Consistent with the lack of a direct biochemical interaction, LegC7 did not disrupt the overall localization of GFP-Vps27 (Fig. 4D, S5B Fig.).

Interestingly, LegC7 expression in the *vps27∆* background did not induce the fragmented punctate pattern of GFP-CPS localization seen previously (Fig. 2A), suggesting that LegC7 may function after Vps27p, as LegC7 is unable to induce additional trafficking defects downstream of the relevant effects imparted by the deletion of *vps27*. Therefore, LegC7 appears to be directly involved in altering protein traffic via the endosome-MVB-vacuole route.

## Discussion

In order to survive intracellularly, *Legionella* separates the LCV from the standard endosomal maturation pathway thus avoiding LCV-lysosome fusion [5]. To this end, *Legionella* secretes a number of effector proteins that appear to directly manipulate endolysosomal compartments. For example, VipD misregulates the early endosomal Rab-family GTPase, Rab5, to promote intracellular survival of the bacterium [49, 50]. Our lab has also characterized another *Legionella* coiled coil containing protein, LegC3, that causes vacuolar fragmentation upon expression in yeast and prevents homotypic vacuole fusion *in vitro* pointing to this protein’s probable role in manipulating host endolysosomal pathways [12]. Due to the importance of separating the LCV from the endosomal pathway and *Legionella’*s broad host range we speculate that other uncharacterized *Legionella* effectors also function to manipulate different aspects of host endosomal systems.

When expressed in yeast, LegC7 disrupts biosynthetic vacuole-directed cargo that emanate from the Golgi, such as CPS and Sna3p. In both cases, the predominant phenotype consists of numerous punctate structures that localize to the cell periphery. Because these proteins are trafficked via similar mechanisms, we suspect that both GFP-CPS and Sna3-GFP are accumulating in the same physiological compartments; perhaps early endosomes that are unable to either mature or fuse to downstream compartments. In addition, by following fluid-phase endocytosis with the soluble dye Lucifer Yellow, we find that yeast cells expressing LegC7 accumulate this marker within the cytosol. Therefore, LegC7 does not completely prevent endocytosis, as the dye is still able to enter the cell, but the LY-containing endosomes fail to deliver their cargo to the vacuole. Interestingly, strains deleted for *vps21* and *ypt52*, the major Rab-family GTPases of the early endocytic pathway, are also reported to display a similar LY accumulation phenotype [51]; this phenotypic similarity to LegC7-expressing strains further suggests that LegC7 may be capable of modulating the early endocytic pathway. It is unknown, however, whether LegC7 directly manipulates these Rab GTPases or the fusion events they catalyze, and therefore requires further study. Upon LegC7 expression, the fluorescent styryl dye, FM4–64 was not seen to accumulate in yeast vacuole membranes, but instead was contained within punctate structures around the cell periphery. The localization pattern observed with FM4–64 mirrors the aberrant accumulation of GFP-CPS and Sna3-GFP in LegC7-expressing cells, leading us to hypothesize that these structures represent the same physiological compartment. Using a GFP tagged version of the a-Factor, Ste3p, we determined that LegC7 also prevented proper vacuolar delivery of receptor mediated endocytic cargoes yet did not disrupt the actual endocytic event.

Our data indicates that LegC7 manipulates traffic involving endosomal maturation, however we wondered if the effects of LegC7 were specific to the endosomal system, or rather represented a global disruption of traffic. As the yeast vacuole receives cargo from at least two other pathways we sought to determine if LegC7 disrupted these pathways as well. In order the probe the ALP pathway which moves cargo directly from the late Golgi to the vacuole in an AP-3 dependent manner we utilized GFP-Vam3, a well-characterized vacuolar SNARE that is known to traffic through the ALP pathway. Localization of GFP-Vam3 was not disrupted by LegC7 expression, nor was the processing of a Cvt-delivered protein, Ape1p. These data indicate that LegC7 specifically disrupts cargo that is required to traffic through endosomes to vacuoles, while not disrupting global cellular trafficking events.

Mutation of the asparagine 242 of LegC7 to either isoleucine or leucine results in a non-toxic derivative of LegC7 that also lacks the endocytic disruption phenotypes of wild type LegC7. Based on *in silico* calculations, this residue is predicted to fall in the very beginning of the putative second coiled-coil domain of LegC7. Furthermore, models predict that replacing this residue with a large aliphatic amino acid alters the predicted linking region between coiled coil region 1 and 2, suggesting that the presence of this domain is critical for proper LegC7 folding or function *in vivo*. In support of these data, early studies with LegC7 found that large deletions of this central coiled-coil domain produced a non-toxic protein, and that the C-terminal coiled-coil domain of LegC7 was not important for toxicity [19]. As the elimination of LegC7 toxicity in our study is fairly specific, we suspect that significant structural changes are induced in LegC7^N242I^, but will not be fully appreciated until crystallographic data are obtained. Recently, the N-terminal portion of a related *Legionella* effector protein, LegC3, was crystallized, resulting in a structure that did not share close homology with any currently known structure [52]. As this crystal structure did not match *in silico* predictions, the structure of LegC7 may provide a new role for the N242 residue in LegC7 function.

Finally, we find that deletions of *VPS27*, and ESCRT-0 complex member, partially reversed the toxic effects of *LEGC7* expression. This effect is not the result of mislocalization of LegC7, but could be explained by the reduction of LegC7 levels through enhanced proteolytic turnover or reduced LegC7 expression in *vps27∆* backgrounds; direct interactions between Vps27p and LegC7 *in vitro* were not detected. Furthermore, we were unable to detect any suppression of LegC7 toxicity in *hse1∆* deletions, which may rule out a function of the intact ESCRT-0 complex in this reversal. It is also possible that Vps27p recruits either a secondary protein required for LegC7 function *in vivo*, or Vps27p plays an as yet undescribed role in an endosomal maturation pathway that LegC7 can exploit. There is a hypothesized link between the ESCRT pathway, which removes membrane surface area of the MVB, and the endocytic fusion pathway, which increases the surface area of the MVB [53, 54]. Perhaps Vps27p, with the earliest function in the yeast ESCRT pathway, serves a role in promoting endosomal fusion or maturation to ensure sufficient surface area of the MVB for proper downstream ESCRT function. It is clear, however, that no other class E protein activity is required for LegC7 toxicity or localization, and we therefore do not believe that LegC7 is directly modulating overall ESCRT function.

The modulation of host endosomal traffic would likely be an important goal for *Legionella*, in both its attempt to evade the normal host endomembrane system, and in the construction of the LCV during infection. It should be noted that *Legionella* strains lacking LegC7 are not defective in macrophage proliferation studies [19], and therefore LegC7-specific activities during *Legionella* infection remain unclear. Identification of the yeast target protein(s) of LegC7 will likely provide essential insight into the role of this effector protein during the intracellular lifecycle of *Legionella*.

## Supporting Information

S1 Fig Quantification of Fig. 2 Microscopy. (A) BY4742 yeast strains harboring GFP-CPS and either the vector control, *LEGC7* ^+^, or *LEGC7* ^*N242I*^ plasmids were grown in selective media supplemented with 2% glucose at 30°C, washed in ddH_2_O, suspended in fresh CSM-uracil-lysine/2% galactose, incubated at 30°C for 16 h, then visualized. (B) BY4742 yeast strains containing GFP-Sna3 and either the vector control, *LEGC7* ^+^, or *LEGC7* ^*N242I*^ plasmids were grown as in (A), then visualized. (C) Cells containing the vector control, *LEGC7* ^+^, or *LEGC7* ^*N242I*^ plasmids were incubated with Lucifer Yellow (Materials and Methods), and then visualized. (D) Strains from (C) were grown in selective media supplemented with 2% glucose at 30°C, washed in ddH_2_O, suspended in fresh CSM-uracil/2% galactose, incubated at 30°C for 16 h, then stained with the yeast vacuolar marker FM4–64 [57] and visualized. (E) Wild type SEY6210 or BWY3400 (∆4+ENTH, Table 1) strains harboring Ste3-GFP and either the vector control or *LEGC7* ^+^ plasmids were grown as in (A) and then visualized. Two separate trials, each consisting of a minimum of 222 individual cells were counted for each set, and images presented are lower magnification/larger fields of those presented in Fig. 2. * *P*<.0261, **P <.0051, *** *P*<.0008, **** *P*<.0001, unpaired two-tailed t Test.
(TIF)

S2 Fig LegC7 does not induce cell death during expression or alter GFP-Vam3 traffic. (A) BY4742 cells containing either a control or *LEGC7* ^+^ plasmid were grown in selective media supplemented with 2% glucose at 30°C, washed in ddH_2_O, suspended in fresh CSM-uracil/2% galactose, and incubated at 30°C for 16 h. A sample of the BY4742 cells were incubated at 100°C for 10 min for a dead cell control, then 25 μM propidium iodide was added, incubated at 30°C for 30 minutes, washed, and visualized. At least 385 cells from each sample were scored for propidium iodide retention; representative micrographs for each condition are shown. (B) Wild type yeast strains expressing GFP-Vam3 [58] and expressing either *LEGC7* ^+^ or *LEGC7* ^*N242I*^ were grown in selective media supplemented with 2% glucose at 30°C, washed in ddH_2_O, suspended in fresh CSM-uracil-lysine/2% galactose, incubated at 30°C for 16 h, then visualized. Two separate trials, each consisting of a minimum of 300 individual cells were counted. n.s.; not significant, unpaired two-tailed t Test. Images presented are lower magnification/larger fields of those presented in Fig. 3.
(TIF)

S3 Fig Most Class E VPS mutants do not reverse LegC7 toxicity. BY4742 or noted class E deletion strains harboring either the control or *LEGC7* ^+^ plasmids were spotted onto CSM-Ura plates containing 2% glucose or 2% galactose in 10-fold serial dilutions (starting OD_600_ = 1.0) and grown at 30°C for 96 h. .
(TIF)

S4 Fig Effects of *vps27∆* on LegC7 function *in vivo*. (A) BY4742 or *vps27∆* strains expressing GFP or GFP-LegC7 were grown in selective media supplemented with 2% glucose at 30°C, stained with FM4–64, and visualized for GFP and FM4–64 fluorescence. (B) BY4742 or *vps27∆* strains harboring either the control or *LEGC7* ^+^ plasmids were grown in selective media containing 2% glucose at 30°C, washed in ddH_2_O, suspended in CSM-Ura 2% galactose, incubated at 30°C for 16 h in order to induce LegC7 expression. Samples were diluted to OD_600_ = 1.0, and cycloheximide was added to a final concentration of 0.5 mg/ml. Cultures were incubated at 30°C and 1 OD of cells were withdrawn at the noted timepoints, processed to extract proteins [55], and separated using SDS-PAGE and immunoblotted using LegC7 antiserum, Sec17 antiserum, or Sec18 (Rabbit 1:1000) serum[59]. Yeast *vps27∆* strains expressing either (C) GFP-CPS or (D) Sna3-GFP harboring the *LEGC7* ^+^ expression plasmid or vector control were grown in selective media supplemented with 2% glucose at 30°C, washed in ddH_2_O, suspended in fresh CSM-uracil-lysine/2% galactose, incubated at 30°C for 16 h, then visualized. Two separate trials, each consisting of a minimum of 214 individual cells were counted for microscopy. ***P<0.002, n.s.=not significant, unpaired two-tailed t Test. Images presented are lower magnification/larger fields of those presented in Fig. 4.
(TIF)

S5 Fig LegC7ΔTM does not interact with GST-Vps27. (A) 3μM LegC7ΔTM was mixed with equimolar concentrations of GST or GST-Vps27 in 20 mM HEPES-NaOH pH 8.0, 10% glycerol, 150mM NaCl, 2mM MgCl2, 1 mM PMSF, and 1 x protease inhibitor cocktail (Pierce). Samples were incubated at 4°C with mixing for 1 hour and input controls were removed. 25μL of equilibrated glutathione resin was added and samples were incubated with mixing for 1 h at 4°C. Samples were washed 10 times with 1 ml of above buffer, suspended in 100μL SDS-PAGE buffer, boiled, and separated via SDS-PAGE. (B) Cells expressing GFP-Vps27 and *LEGC7* ^+^ were grown in selective media supplemented with 2% glucose at 30°C, washed in ddH_2_O, suspended in fresh CSM-uracil-lysine/2% galactose, incubated at 30°C for 16 h, then visualized. Two separate trials consisting of a minimum of 300 individual cells were counted; n.s.=not significant; unpaired two-tailed t Test. Images presented are lower magnification/larger fields of those presented in Fig. 4.
(EPS)
