# Supplementary figures and images for: The Legionella pneumophila Effector Protein, LegC7, Alters Yeast Endosomal Trafficking
Source: PLoS One. 2015 Feb 2;10(2):e0116824. doi: 10.1371/journal.pone.0116824 (PMC4314205; doi:10.1371/journal.pone.0116824)

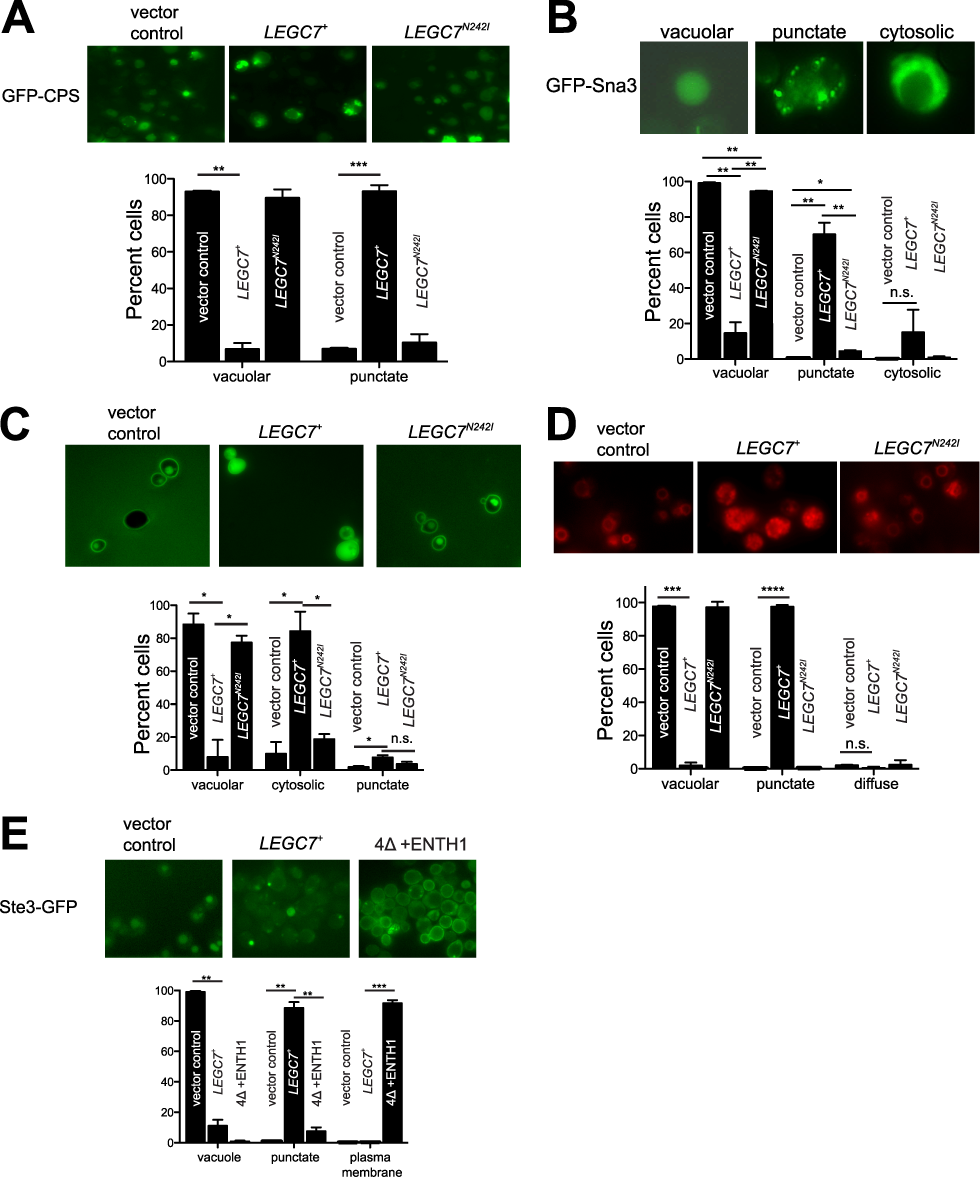

Supplement: S1 Fig — (A) BY4742 yeast strains harboring GFP-CPS and either the vector control, LEGC7 +, or LEGC7 N242I plasmids were grown in selective media supplemented with 2% glucose at 30°C, washed in ddH2O, suspended in fresh CSM-uracil-lysine/2% galactose, incubated at 30°C for 16 h, then visualized. (B) BY4742 yeast strains containing GFP-Sna3 and either the vector control, LEGC7 +, or LEGC7 N242I plasmids were grown as in (A), then visualized. (C) Cells containing the vector control, LEGC7 +, or LEGC7 N242I plasmids were incubated with Lucifer Yellow (Materials and Methods), and then visualized. (D) Strains from (C) were grown in selective media supplemented with 2% glucose at 30°C, washed in ddH2O, suspended in fresh CSM-uracil/2% galactose, incubated at 30°C for 16 h, then stained with the yeast vacuolar marker FM4–64 [57] and visualized. (E) Wild type SEY6210 or BWY3400 (∆4+ENTH, Table 1) strains harboring Ste3-GFP and either the vector control or LEGC7 + plasmids were grown as in (A) and then visualized. Two separate trials, each consisting of a minimum of 222 individual cells were counted for each set, and images presented are lower magnification/larger fields of those presented in Fig. 2. * P<.0261, **P <.0051, *** P<.0008, **** P<.0001, unpaired two-tailed t Test. (TIF) [file pone.0116824.s001.tif]

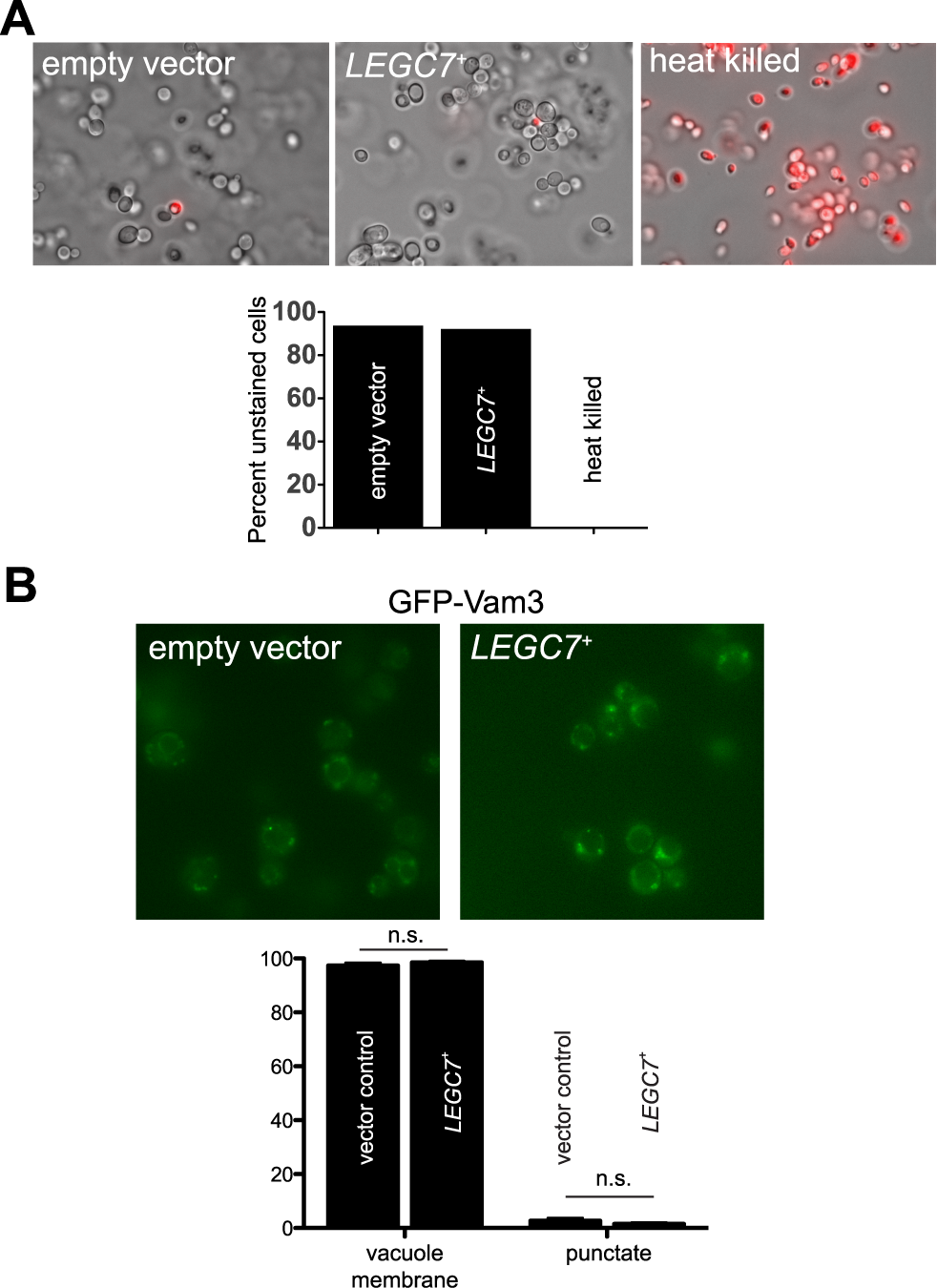

Supplement: S2 Fig — (A) BY4742 cells containing either a control or LEGC7 + plasmid were grown in selective media supplemented with 2% glucose at 30°C, washed in ddH2O, suspended in fresh CSM-uracil/2% galactose, and incubated at 30°C for 16 h. A sample of the BY4742 cells were incubated at 100°C for 10 min for a dead cell control, then 25 μM propidium iodide was added, incubated at 30°C for 30 minutes, washed, and visualized. At least 385 cells from each sample were scored for propidium iodide retention; representative micrographs for each condition are shown. (B) Wild type yeast strains expressing GFP-Vam3 [58] and expressing either LEGC7 + or LEGC7 N242I were grown in selective media supplemented with 2% glucose at 30°C, washed in ddH2O, suspended in fresh CSM-uracil-lysine/2% galactose, incubated at 30°C for 16 h, then visualized. Two separate trials, each consisting of a minimum of 300 individual cells were counted. n.s.; not significant, unpaired two-tailed t Test. Images presented are lower magnification/larger fields of those presented in Fig. 3. (TIF) [file pone.0116824.s002.tif]

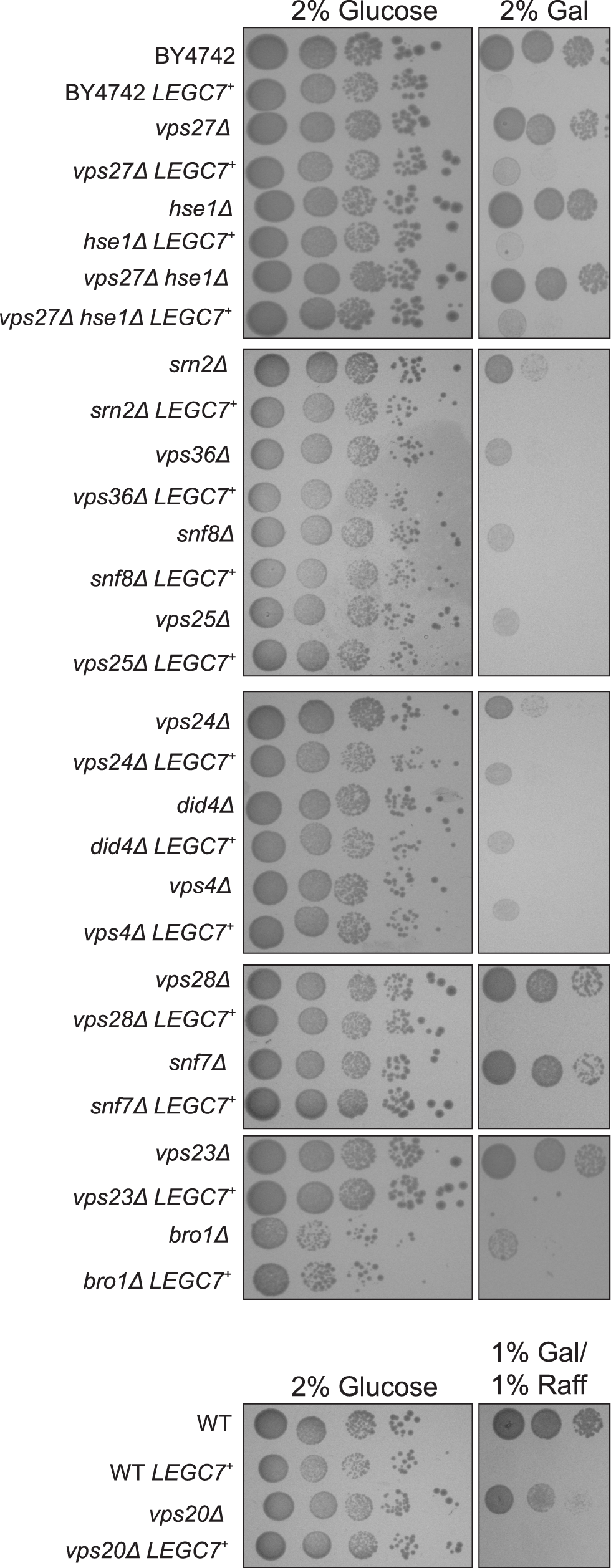

Supplement: S3 Fig — BY4742 or noted class E deletion strains harboring either the control or LEGC7 + plasmids were spotted onto CSM-Ura plates containing 2% glucose or 2% galactose in 10-fold serial dilutions (starting OD600 = 1.0) and grown at 30°C for 96 h. . (TIF) [file pone.0116824.s003.tif]

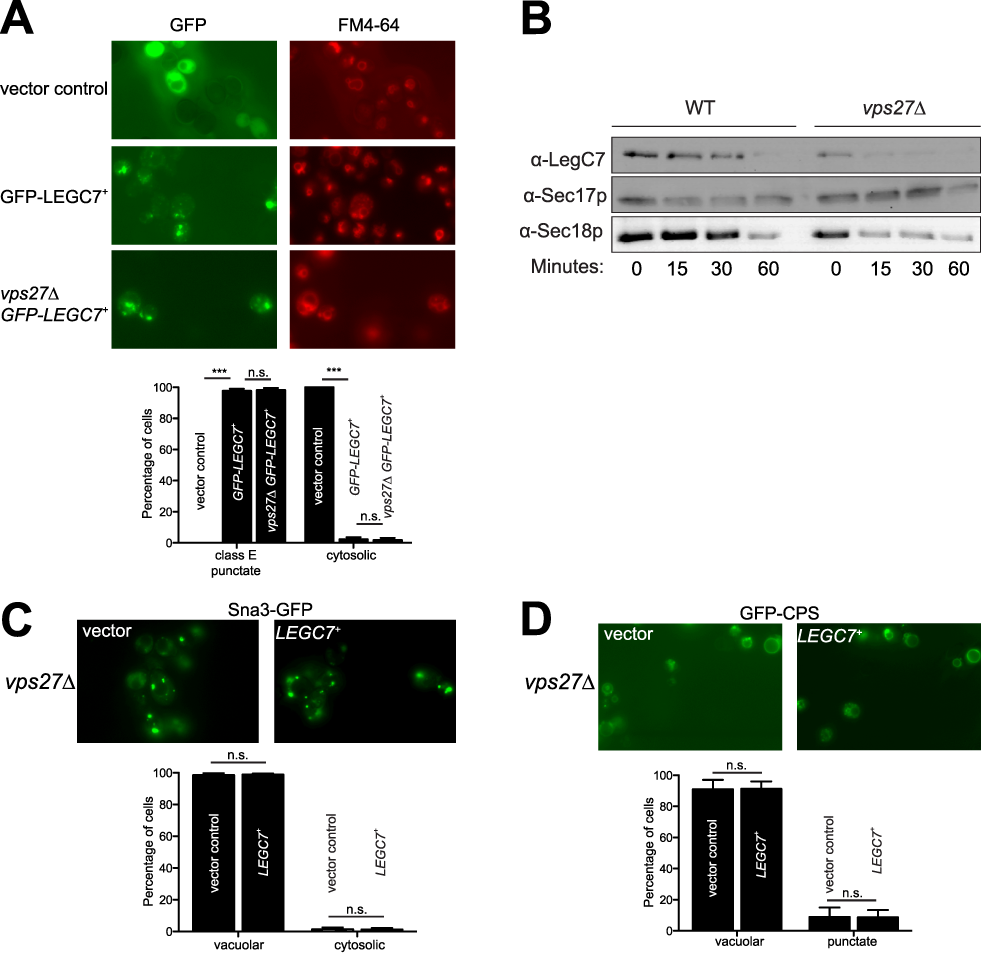

Supplement: S4 Fig — (A) BY4742 or vps27∆ strains expressing GFP or GFP-LegC7 were grown in selective media supplemented with 2% glucose at 30°C, stained with FM4–64, and visualized for GFP and FM4–64 fluorescence. (B) BY4742 or vps27∆ strains harboring either the control or LEGC7 + plasmids were grown in selective media containing 2% glucose at 30°C, washed in ddH2O, suspended in CSM-Ura 2% galactose, incubated at 30°C for 16 h in order to induce LegC7 expression. Samples were diluted to OD600 = 1.0, and cycloheximide was added to a final concentration of 0.5 mg/ml. Cultures were incubated at 30°C and 1 OD of cells were withdrawn at the noted timepoints, processed to extract proteins [55], and separated using SDS-PAGE and immunoblotted using LegC7 antiserum, Sec17 antiserum, or Sec18 (Rabbit 1:1000) serum[59]. Yeast vps27∆ strains expressing either (C) GFP-CPS or (D) Sna3-GFP harboring the LEGC7 + expression plasmid or vector control were grown in selective media supplemented with 2% glucose at 30°C, washed in ddH2O, suspended in fresh CSM-uracil-lysine/2% galactose, incubated at 30°C for 16 h, then visualized. Two separate trials, each consisting of a minimum of 214 individual cells were counted for microscopy. ***P<0.002, n.s.=not significant, unpaired two-tailed t Test. Images presented are lower magnification/larger fields of those presented in Fig. 4. (TIF) [file pone.0116824.s004.tif]
